# Lineage-Specific Restraint of Pituitary Gonadotroph Cell Adenoma Growth

**DOI:** 10.1371/journal.pone.0017924

**Published:** 2011-03-25

**Authors:** Vera Chesnokova, Svetlana Zonis, Cuiqi Zhou, Anat Ben-Shlomo, Kolja Wawrowsky, Yoel Toledano, Yunguang Tong, Kalman Kovacs, Bernd Scheithauer, Shlomo Melmed

**Affiliations:** 1 Department of Medicine, Pituitary Center, Cedars-Sinai Medical Center, Los Angeles, California, United States of America; 2 Departments of Pathology, St. Michael's Hospital, Toronto, Canada; 3 Mayo Clinic, Rochester, Minnesota, United States of America; Consiglio Nazionale delle Ricerche (CNR), Italy

## Abstract

Although pituitary adenomas are usually benign, unique trophic mechanisms restraining cell proliferation are unclear. As GH-secreting adenomas are associated with p53/p21-dependent senescence, we tested mechanisms constraining non-functioning pituitary adenoma growth. Thirty six gonadotroph-derived non-functioning pituitary adenomas all exhibited DNA damage, but undetectable p21 expression. However, these adenomas all expressed p16, and >90% abundantly expressed cytoplasmic clusterin associated with induction of the Cdk inhibitor p15 in 70% of gonadotroph and in 26% of somatotroph lineage adenomas (p = 0.006). Murine LβT2 and αT3 gonadotroph pituitary cells, and αGSU.*PTTG* transgenic mice with targeted gonadotroph cell adenomas also abundantly expressed clusterin and exhibited features of oncogene-induced senescence as evidenced by C/EBPβ and C/EBPδ induction. In turn, C/EBPs activated the clusterin promoter ∼5 fold, and elevated clusterin subsequently elicited p15 and p16 expression, acting to arrest murine gonadotroph cell proliferation. In contrast, specific clusterin suppression by RNAis enhanced gonadotroph proliferation. FOXL2, a tissue-specific gonadotroph lineage factor, also induced the clusterin promoter ∼3 fold in αT3 pituitary cells. As nine of 12 pituitary carcinomas were devoid of clusterin expression, this protein may limit proliferation of benign adenomatous pituitary cells. These results point to lineage-specific pathways restricting uncontrolled murine and human pituitary gonadotroph adenoma cell growth.

## Introduction

Pituitary tumors arise from highly specialized cell types expressing the respective pituitary polypeptide hormones. Thus, tumors derived from somatotrophs secrete growth hormone (GH), lactotrophs, prolactin (PRL), thyrotrophs, thyrotropin (TSH), and corticotrophs, adrenocorticotropin (ACTH). In contrast, non-functioning pituitary tumors usually arise from non-secreting cells of gonadotroph origin [1]. Clinically inapparent pituitary tumors are identified in 25% of autopsy specimens with a population prevalence of ∼77 cases/10^5^. Pituitary tumors are usually benign neoplasms (adenomas), however, they may also exhibit invasive or recurrent growth. Rarely encountered malignant pituitary carcinomas comprise 0.02% of all pituitary tumors, proliferate rapidly and show extracranial metastases [2], [3], [4]. Although most aggressive pituitary adenomas persistently exhibit low mitotic activity [3], mechanisms underlying these unique growth properties are largely elusive. We postulate that intrinsic cell-specific trophic properties as well as the lineage-origin of highly differentiated and specialized pituitary cells underlies constrained adenoma proliferation.

Cellular senescence is characterized by irreversible proliferative arrest, while cells remain viable and metabolically active. Proliferation arrest may occur as a result of age-related telomere shortening, and also in response to oxidative or genotoxic stress, DNA damage, aneuploidy or chromosomal instability, as well as oncogene activation [5], [6]. Thus, oncogenic RAS causes stable proliferative arrest rather than transformation in diploid fibroblasts [7]. BRAF in benign skin nevi elicits an initial increased proliferation followed by DNA stress and cellular senescence [8]. As cellular senescence appears to be bypassed in advanced malignancies [8], [9], [10], this antiproliferative mechanism may represent an initial impediment against oncogenic development [11]. Senescence is mediated by activation of p53/p21 and other Cdk inhibitors including p15 and p16 [5]. Oncogene-inuced senescence is also associated with a senescence-messaging secretome, enabling senescence responses [11].

Clusterin (CLU), a highly conserved cellular and circulating protein [12], is also known as apolipoprotein J (ApoJ), sulphated glycoprotein 2 ( SGP-2), testosterone-repressed prostate message 2 (TRPM-2), or serum protein-40(SP-40). Intracellular clusterin forms include a partially glycosylated uncleaved pre-secretory protein, a secretory/intracellular glycosylated α-β chain heterodimer, and a anti-apoptotic nuclear form [13], [14]. Nuclear clusterin is induced in response to DNA damage evoked by chemotherapeutic agents, and protects some tumorous cells from apoptosis [15], [16]. In contrast, intracellular clusterin was also shown to inhibit prostate cancer cell proliferation, and clusterin knockout induced highly aggressive transgenic mouse prostate tumors [17]. Clusterin thus functions as a tumor suppressor gene [18], inhibits cell proliferation [19], [20], promotes experimental skin carcinoma differentiation [21], and exhibits features of a cellular stress responder [14].

Pituitary tumor transforming gene (*PTTG*) induction is a hallmark of human pituitary tumors [22], [23], [24]. PTTG was isolated from pituitary tumor cells [25] and PTTG abundance also correlates with breast, thyroid, endometrial, esophageal and colorectal tumor invasiveness [26]. PTTG facilitates cell cycle progression [22], [25] and when over-expressed, causes cell transformation [22] and promotes tumor formation *in vivo* [27]. Transgenic *PTTG* over-expression targeted to pituitary gonadotroph cells results in focal pituitary adenoma formation [28], while *Pttg* deletion abrogates murine pituitary tumor development [29]. PTTG is the index mammalian securin [30], and both *Pttg* deletion [31] as well as overexpression [27] result in aneuploidy and chromosomal instability highlighting the requirement for intracellular securin equilibrium to maintain chromosomal stability [32], [33].

In most human GH-producing pituitary adenomas PTTG overexpression is associated with DNA damage and p21-dependent senescence [34], however pathways restraining growth and transformation of the more commonly encountered non-functioning pituitary adenomas are not known. We show here that similar to GH-cell adenomas, tumors arising from the gonadotroph lineage exhibit high PTTG levels and DNA damage. However, unlike GH-cell adenomas, p53/p21 senescence markers are not activated in non-functioning adenomas, which do, however, selectively express abundant cytoplasmic clusterin. High clusterin levels restrain cell proliferation by triggering Cdk inhibitors p15, p16 and p27, while suppression of clusterin expression enhanced pituitary gonadotroph cell proliferation. Thus, we identify a novel role for clusterin in enabling pituitary gonadotroph tumor cell proliferation arrest. FOXL2, a transcription factor specifically expressed in pituitary gonadotroph cells [35] stimulates the clusterin promoter, further highlighting a differential lineage-specific pathway restricting pituitary cell cycle progression, acting to buffer non-functioning pituitary adenomas from unrestrained growth.

## Results

### DNA damage and senescence markers are induced in human pituitary adenomas

**Figure 1:**
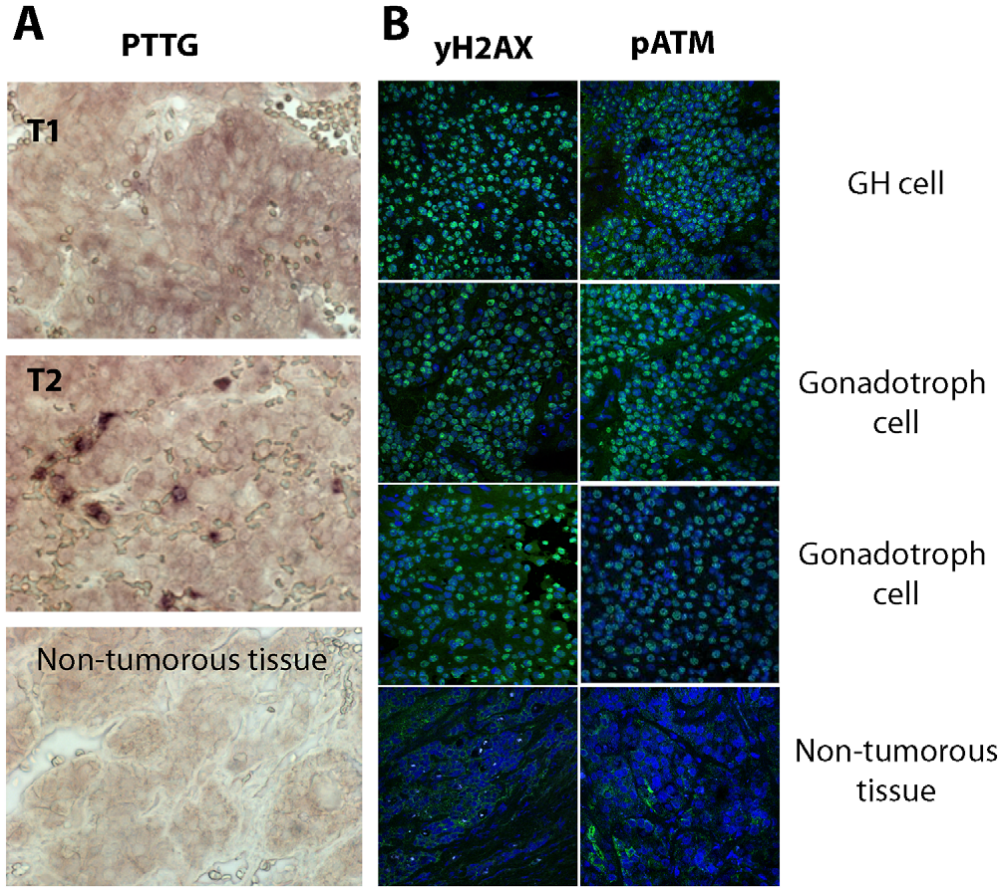
PTTG and DNA damage in human pituitary adenomas. **A**) PTTG immunoreactivity (brown signal, both intranuclear and cytoplasmic)) in non-tumorous pituitary and human pituitary gonadotroph adenomas (T-1,2); **B**) DNA damage in human pituitary adenoma. Confocal image of human pituitary adenoma and non-tumorous pituitary tissue specimens labeled with γH2.AX or pATM antibodies (green). Both proteins are expressed in the nucleus. Specimens here and shown below were counterstained with DNA-specific dye ToPro3 (blue).

Immunoreactive PTTG was induced in all 36 gonadotroph cell adenomas analyzed, but not in normal pituitary tissue (Figure 1A), confirming previous reports [22], [23], [24]. Markers of DNA damage and aneuploidy including γH2A.X foci and phopsphorylated kinase mutated in ataxia telangiectasia (pATM) [36], [37] were not detected by fluorescent immunohistochemistry in two non-tumorous human pituitary specimens. In contrast, all of 12 human pituitary adenomas analyzed (2 GH cell, 10 non-secreting gonadotroph cell) invariably expressed both γH2A.X and pATM, reflecting activated DNA damage signaling (Figure 1B).

**Figure 2:**
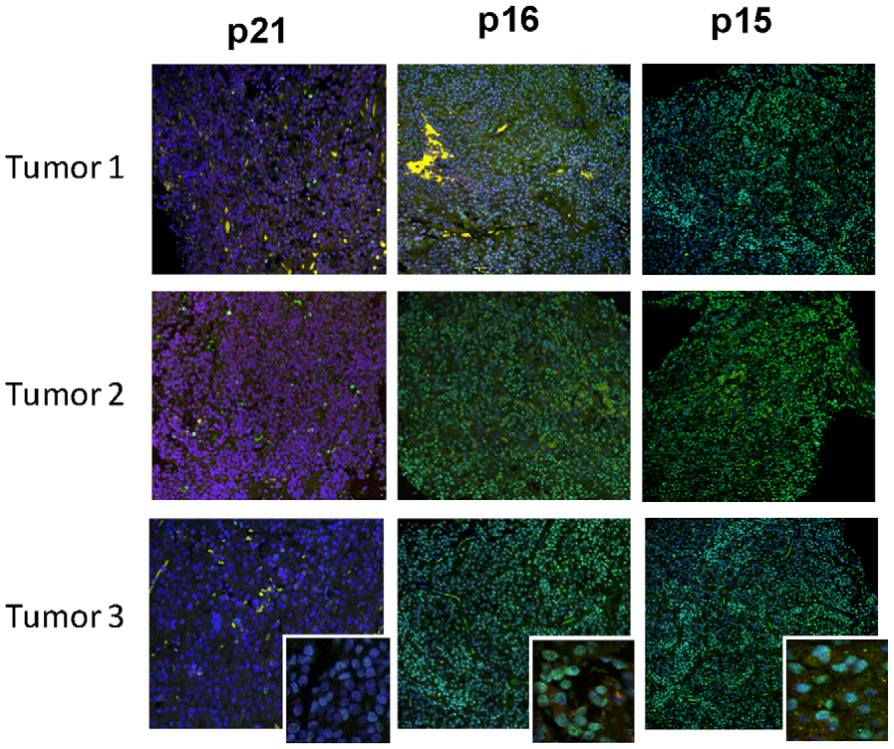
Cdk inhibitor expression in human pituitary adenomas. Confocal image of human gonadotroph pituitary adenoma specimens labeled with p21, p16 or p15 antibodies (green, intranucelar).

We showed earlier that human GH-secreting adenomas, but not carcinomas, abundantly express intra-nuclear p21, an end-point inhibitor of cell proliferation in the senescence pathway [29], [34]. In contrast, gonadotroph cell adenomas did not express p21 (Figure 2). However, because these tumors also do not (with exceedingly rare exceptions) evolve to malignancy, we analyzed additional pathways restraining pituitary tumor cell proliferation. p16 and p15 of the ARF/INK Cdk inhibitor family act to restrain cellular proliferation in response to activated oncogenes [8], [10], and were strongly expressed in gonadotroph adenomas. Seventy percent of gonadotroph adenomas expressed high levels of p15, as compared to 26% of GH-secreting adenomas (p = 0.006, Table 1). Thus, both DNA damage pathways and senescence markers were expressed in gonadotroph cell-derived adenomas (Figure 2).

**Table 1:** p15 and clusterin expression in human pituitary adenomas.

		p15 n (%)				Clusterin n (%)		
Intensity (%)		0–5	6–50	51–100		0–5	6–50	51–100
Tumor Types	*n*				*n*			
GH/PRL	27	16(59)	4(15)	7(26)	17	13(76)	3(18)	1(6)
Gonadotroph	43	8(18)	5(12)	30(70)	36	1(3)	2(6)	33(92)
ACTH	14	2(14)	1(7)	11(79)	10	1(10)	0(0)	9(90)
Carcinoma	N/A				12	9(76)	2(16)	1(8)
Normal pituitary	5	1(20)	2(40)	2(40)	4	2(50)	2(50)	0(0)

Protein distribution differences between GH/PRL and gonadotroph tumors, p = 0.0006 for p15, and p = 0.0001 for clusterin (Wilcoxon Rank Sum Test). N/A- not available.

### Clusterin is expressed predominantly in gonadotroph cell adenomas

**Figure 3:**
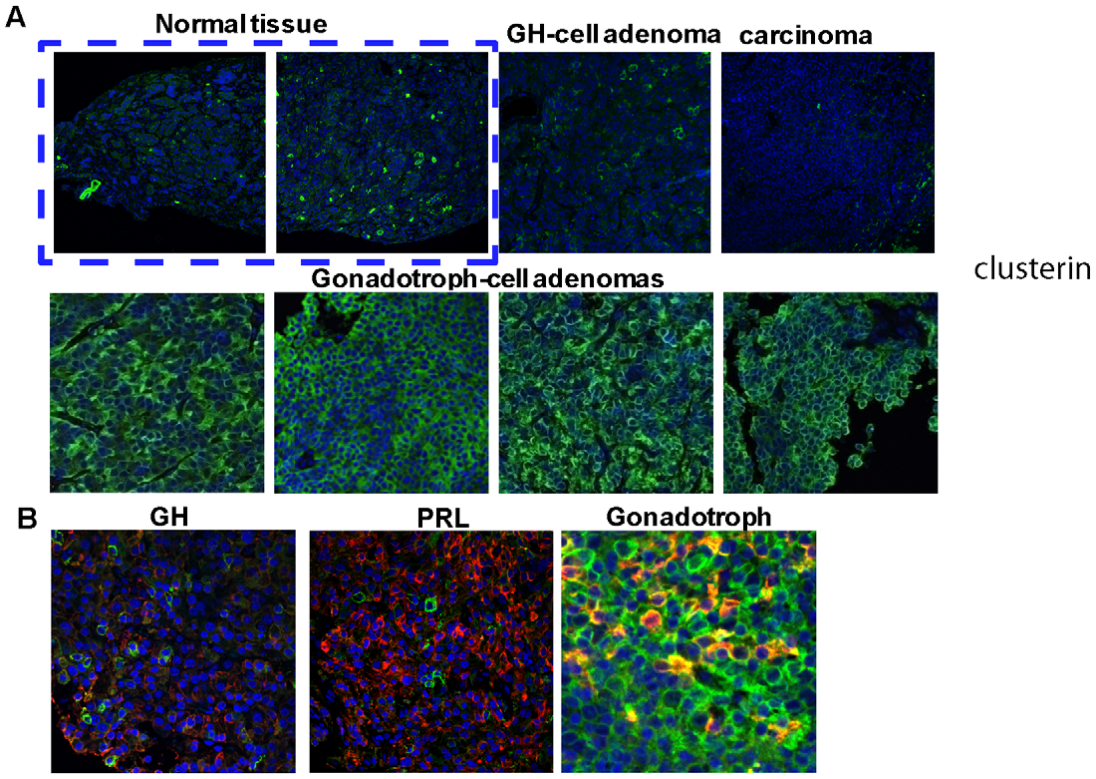
Clusterin in human pituitary adenomas. Confocal image of **A**) human pituitary adenoma and non-tumorous pituitary tissue specimens showing clusterin (green) expressed exclusively in the cytoplasm; **B**) Co-localization of clusterin with GH, PRL and αGSU in respective human pituitary adenoma specimens (clusterin green, respective hormones red).

As Affymetrix datasets (www.oncomine.org) showed that clusterin mRNA expression was low in advanced metastatic cancers [18], we measured clusterin immunofluorescence in non-tumorous pituitary tissue, and in pituitary adenoma cell types (Figure 3). Of 4 non-tumorous pituitary tissues, 2 exhibited intermediate (<50% positivity), while 2 cases exhibited very low (0–5%) clusterin levels. Only 1 of 17 GH/PRL-secreting adenomas (6%) expressed high clusterin levels, 3 expressed moderate levels, while this protein was minimally detectable in 13 such adenomas. In contrast, 33 of 36 (92%) of the more commonly encountered non-functioning adenomas exhibited abundant clusterin cytoplasmic immunopositivity. We also analyzed 12 pituitary carcinomas (9 corticotroph cell and 3 PRL cell ). None of PRL cell carcinomas express clusterin, only 1 corticotroph cell carcinoma (8%) showed high clusterin levels, 2 (16%) exhibited moderate clusterin levels, while clusterin was undetectable in 6 corticotroph cell carcinoma samples. Thus, 9 of 12 (76%) carcinomas did not express clusterin (Figure 3 and Table 1). Three different adenoma types (GH-, PRL- and gonadotroph) were each analyzed for co-localization of clusterin with respective pituitary hormone markers. Moderately expressed clusterin in GH-secreting adenomas did not co-localize with GH, nor with PRL in PRL cell adenomas. In contrast, clusterin strongly co-localized with αGSU in all 3 gonadotroph cell adenomas analyzed (Figure 3B).

### αGSU.PTTG mice exhibit features of oncogene-induced pituitary senescence

**Figure 4:**
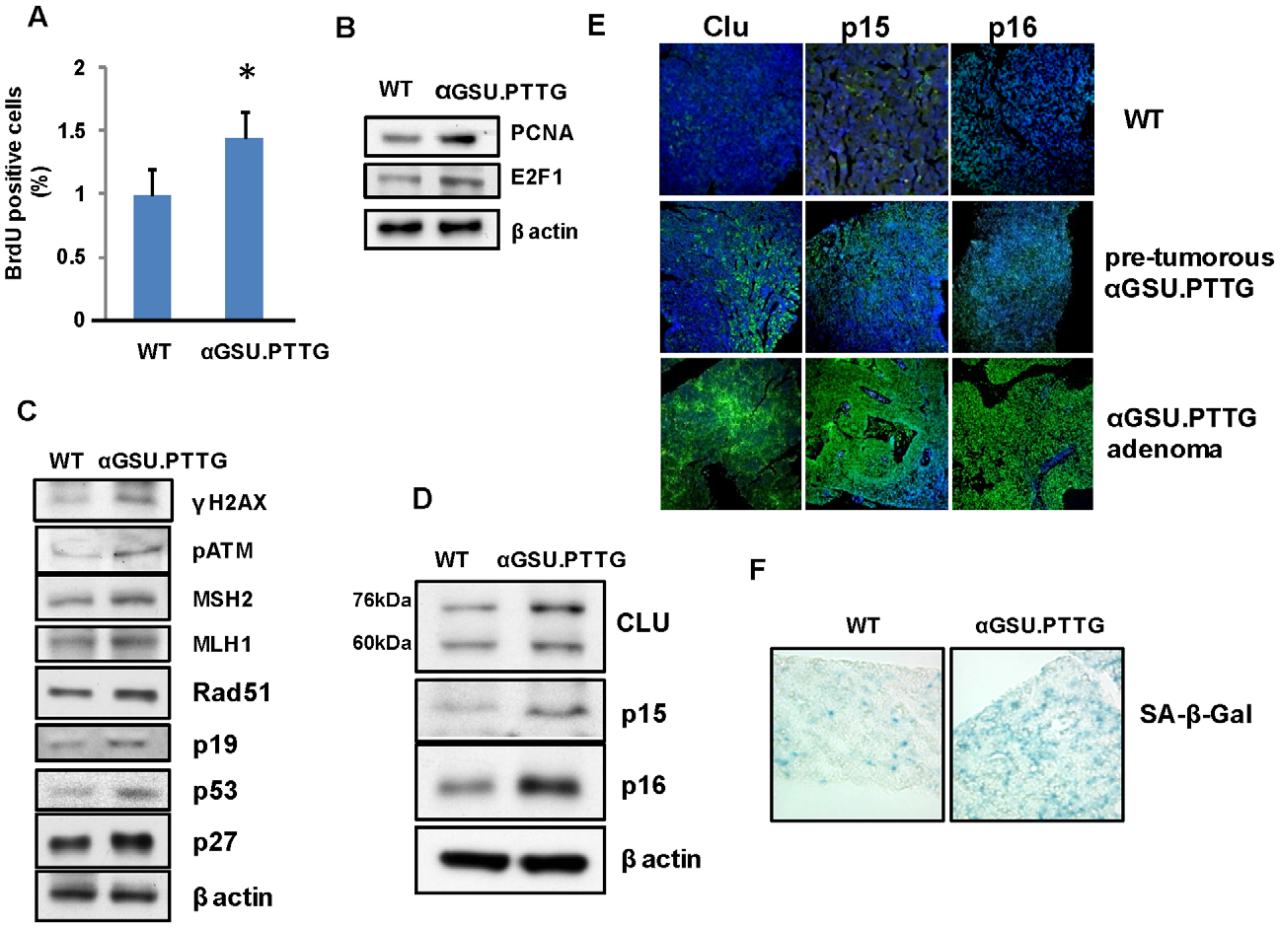
Pituitary proliferation, DNA damage and senescence markers in the αGSU.*PTTG* pituitary gland. **A**) *In vivo* BrdU incorporation. Mice were injected with BrdU (50 µg/g BW), and pituitary sections stained for BrdU. One thousand cells/section, 3 sections/animal, n = 3 animals/genotype were analyzed. *, p<0.05; Western blot analysis of **B**) proliferation markers; **C**) DNA damage, DNA repair and p53-dependent senescence markers, and **D**) oncogene-induced senescence markers; **E**) Confocal image showing immunofluorescent cytoplasmic clusterin, and intranuclear p15 and p16 expression (green) in WT and in pre-tumorous αGSU.*PTTG* pituitary glands, and in αGSU.*PTTG* pituitary adenomas; **F**) Pituitary SA-β-galactosidase enzymatic activity (blue) in WT and in pre-tumorous αGSU.*PTTG* pituitary gland. Three pituitary cryosections/animal were analyzed from 3 animals/genotype, and a representative image shown. Western blots here and elsewhere were repeated 3 times with similar results and representative blots shown.

We recapitulated human gonadotroph tumors in an *in vivo* transgenic murine model of gonadotroph *PTTG* expression driven by the αGSU promoter [28]. αGSU.*PTTG* pituitary glands express up-regulated gonadotroph PTTG with pituitary hyperplasia starting from 4 months of age leading to development of focal pituitary adenomas expressing LH. Other transgenic lines also expressed GH and PRL [38]. In accordance with evidence supporting proto-oncogenic properties of PTTG [26], hyperplastic pre-tumorous pituitary glands derived from transgenic animals were shown to already express markers of increased pituitary proliferation as evidenced by increased BrdU incorporation (Figure 4A ), and elevated levels of pro-proliferative proteins including PCNA and E2F1 in vivo (Figure 4B). However, as these animals developed penetrant pituitary tumors only after 10 months, and these invariably remain small, we tested whether pituitary PTTG overexpression also affects anti-proliferative pathways in these transgenic mice.

DNA damage was already evident in pre-tumorous transgenic pituitary glands overexpressing PTTG as evidenced by enhanced pituitary γH2A.X and pATM levels, accompanied by induced DNA damage repair proteins including MSH2, MLH1 and Rad51, as well as tumor suppressors including p19 and p53 (Figure 4C). The Cdk inhibitor p27, a marker of DNA damage [39], was also induced in pre-tumorous αGSU.*PTTG* pituitary glands, as were the cell cycle suppressor proteins p15 and p16 (Figure 4D). Two intracellular forms of pituitary clusterin, a mature glycosylated ∼76 kDa secretory form and ∼60 kDA pre-secretory form were up-regulated in the pretumorous transgenic pituitary gland (Figure 4D).

These results were confirmed by fluorescent immunostaining. Although only modest cytoplasmic clusterin, and intra-nuclear p15 and p16 expression were observed in WT murine pituitary glands, expression of these 3 proteins was enhanced in the transgenic pre-tumorous pituitary, and further induced in αGSU.*PTTG* pituitary tumors (Figure 4E). Thus, features of oncogene-induced senescence in the αGSU.*PTTG* pituitary included induction of the p19/p53/p27 DNA damage pathway, and both p15 and p16 Cdk inhibitors [7], [40]. In the pre-tumorous hyperplastic αGSU.PTTG pituitary gland the observed increased SA-β galactosidase activity supported the presence of cellular senescence (Figure 4F).

### Pttg over-expression in LβT2 cells results in a senescent phenotype

**Figure 5:**
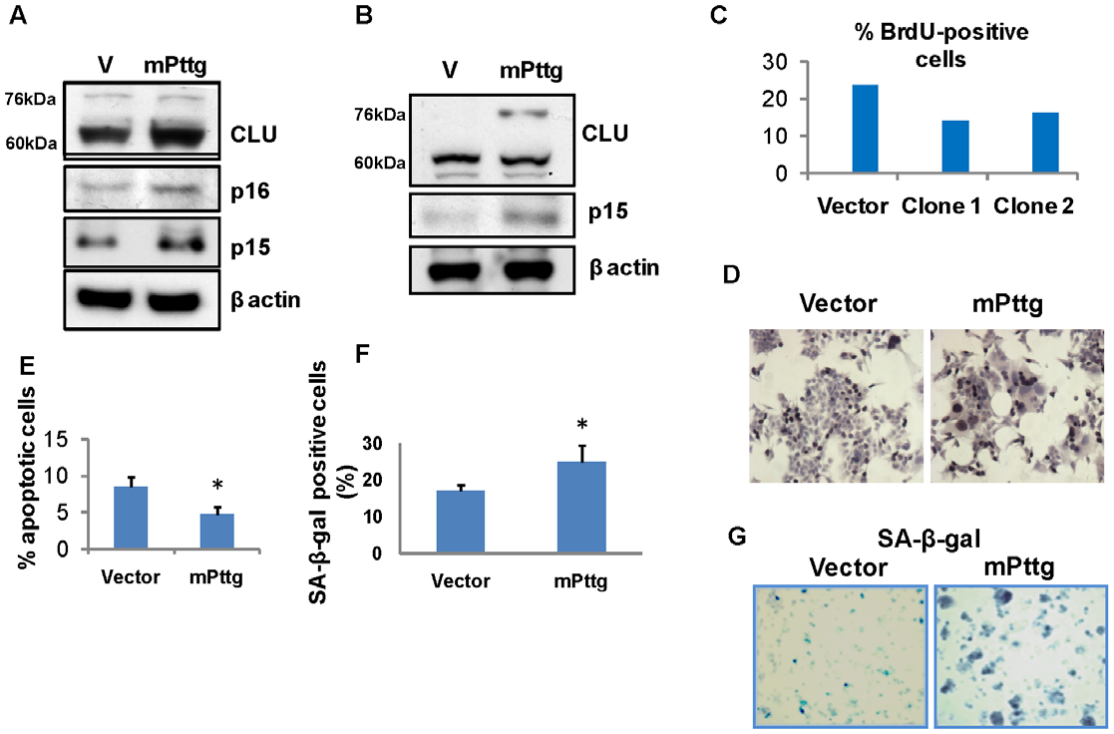
Senescence markers in gonadotroph-derived LβT2 cells transfected with m*Pttg*. Western blot analysis of senescence markers in **A**) LβT2 cells transiently transfected with m*Pttg*; **B**) in LβT2 cells stably transfected with m*Pttg*; **C**) Percent BrdU positive cells in two selected clones stably transfected with m*Pttg*. Duplicate samples were pulsed with BrdU for 30 min and analyzed by flow cytometry; **D**) Senescent morphology of LβT2 cells stably transfected with m*Pttg*. Brown dots depict incorporated BrdU; **E**) Percent apoptotic cells stably transfected with m*Pttg*. Cells were fixed, and one thousand cells/field counted in three randomly chosen visual fields; **F**) Percent SA-β-galactosidase positivity in cells stably transfected with *Pttg* was assessed in 6-well plates in triplicate. One thousand cells/field were counted in three fields/well. **G**) SA-β-galactosidase enzymatic activity (blue) in cells stably transfected with m*Pttg*. *, p<0.05.

To recapitulate *in vivo* effects of pituitary *Pttg* over-expression, we transiently transfected murine gonadotroph-derived LβT2 cells with a plasmid expressing murine *Pttg*, and also isolated LβT2 cells stably overexpressing *Pttg*. As shown in Figure 5, *Pttg* overexpression lead to induction of clusterin and p15 in both gonadotroph cell transfectants, similar to *in vivo* patterns observed in the αGSU.*PTTG* pituitary (Figure 4D).

Clones of stably transfected LβT2 cells sorted and selected for high PTTG expression, showed lower rates of BrdU incorporation as compared to control vector-expressing cells, reflecting decreased proliferation (Figure 5C). These transfectants were spread-out, and larger in size with giant aneuploid nuclei, consistent with a senescent phenotype (Figure 5D). High *Pttg* expression resulted in decreased apoptosis as detected by TUNEL assay (Figure 5E), and these cells also exhibited increased SA-β-galactosidase activity (Figure 5 F,G). Thus, constitutively high gonadotroph cell *Pttg* expression resulted in premature cellular senescence similar to the *in vivo* pituitary phenotype observed in αGSU.*PTTG* mice.

### C/EBPs induce pituitary cell clusterin

**Figure 6:**
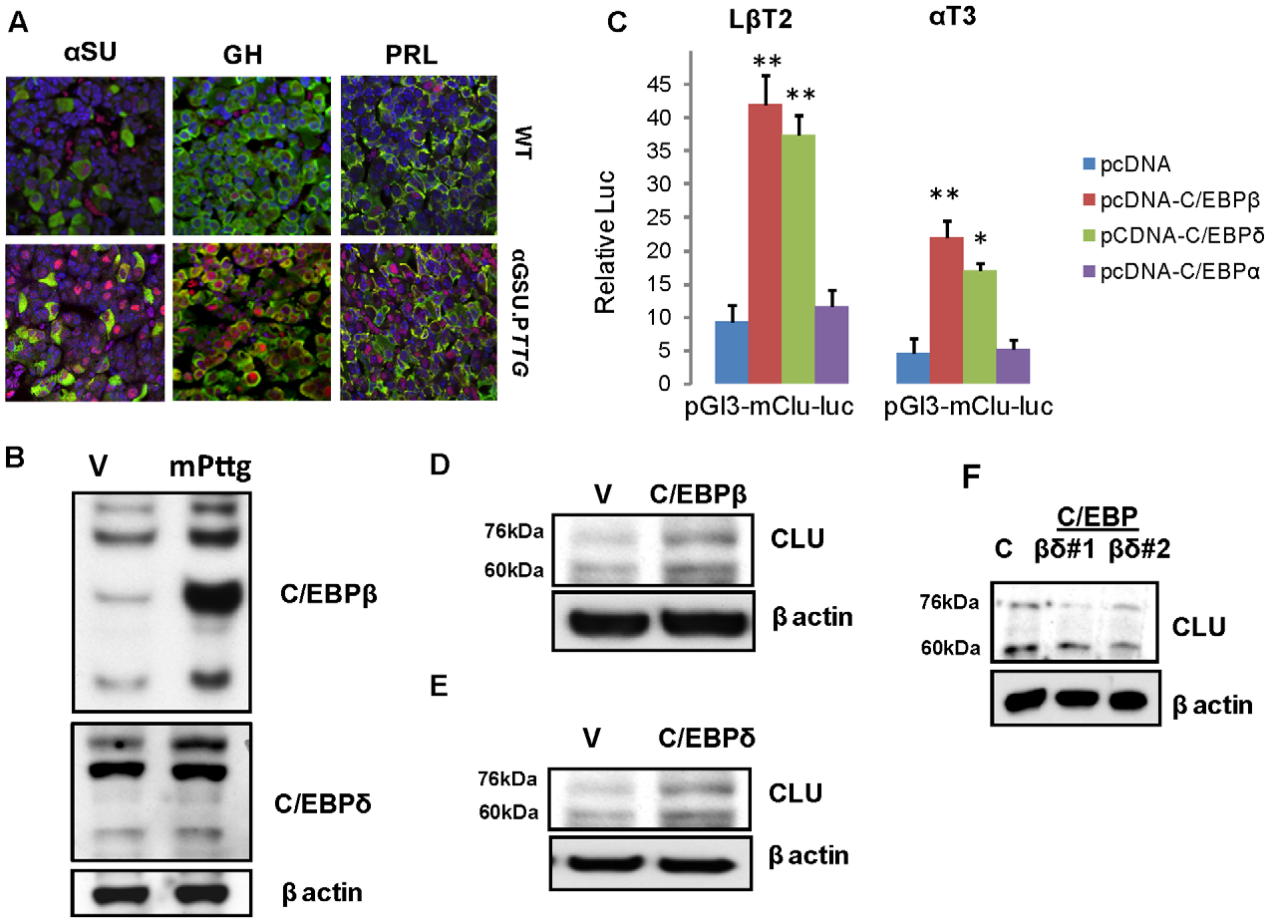
C/EBPs induce clusterin. **A**) C/EBPβ is up-regulated in the αGSU.*PTTG* pituitary. Confocal image showing C/EBPβ co-localization with αGSU-positive, GH-positive and PRL-positive cells in WT and pre-tumorous αGSU.*PTTG* pituitary glands. (Hormones-green, cytoplsmic, C/EBPβ–red, intranuclear); **B**) Western blot analysis of C/EBPβ and δ isoforms induced in LβT2 cells stably transfected with m*Pttg*; **C**) Effects of C/EBPs on the clusterin promoter in LβT2 and αT3 cells 24 h after transfection. Cells were co-transfected with 200 ng murine pGL3-luc-m*Clu* reporter plasmid and 800 ng murine pCDNA3-C/EBPα, β or δ. The ratio of luciferase to co-trasfected β-galactosidase control reporter vector was normalized to pCDNA3-null expression vector. SEM was calculated from triplicate assays, and experiments repeated three times with similar results. Results of a representative experiment are shown.*, p<0.05, **,p<0.01; **D**) Western blot analysis of clusterin expression in gonadotroph-derived αT3 cells 24 hours after transfection with pCDNA3-C/EBPβ or **E**) pCDNA3-C/EBPδ; **F**) Western blot analysis of clusterin expression in LβT2 mPttg cells 48 hours after simultaneous transfection with siC/EBPβ and siC/EBPδ (3 nM each). Two different combinations of siRNAs were used.

C/EBP transcription factors are involved in cellular proliferation and differentiation [41], [42], [43]. In the pre-tumorous hyperplastic αGSU.PTTG pituitary, C/EBPβ was induced both in αGSU, and in GH- and PRL-secreting cells (Figure 6A). In LβT2 cells stably expressing *Pttg*, several C/EBPβ and C/EBPδ isoforms [41] ) were also up-regulated (Figure 6 B).

We therefore assessed whether C/EBPs activate the clusterin promoter in LβT2 cells, and also in a murine gonadotroph-derived αT3 cell line. pGL3-m*Clu*-luc reporter plasmid co-transfected with full length murine C/EBPβ or with C/EBPδ constructs (Figure 6C) resulted in induced luciferase activity in both cell types. However, the plasmid encoding C/EBPα did not induce the clusterin promoter, indicating the specificity of C/EBPβ and δ effects. Accordingly, clusterin protein levels were also found to be up-regulated in cells transfected with pcDNA3-C/EBPβ and δ respectively (Figure 6 D,E).

As LβT2 *mPttg* cells exhibit increased clusterin levels, we treated these cells with siRNAs directed against either C/EBPβ or C/EBPδ. However, separate suppression of either of these genes resulted in compensatory increase of the other protein (data not shown), while simultaneous suppression of C/EBPβ and δ with 3 nM of each RNAi lead to decreased clusterin protein levels in LβT2 *mPttg* cells after 48 hours. These experiments were conducted with two different siRNA combinations directed against both C/EBPβ and δ, and a representative Western blot is shown in Figure 6F. The results confirmed that both C/EBP proteins act to regulate gonadotroph cell clusterin expression.

### Clusterin restrains pituitary cell proliferation

**Figure 7:**
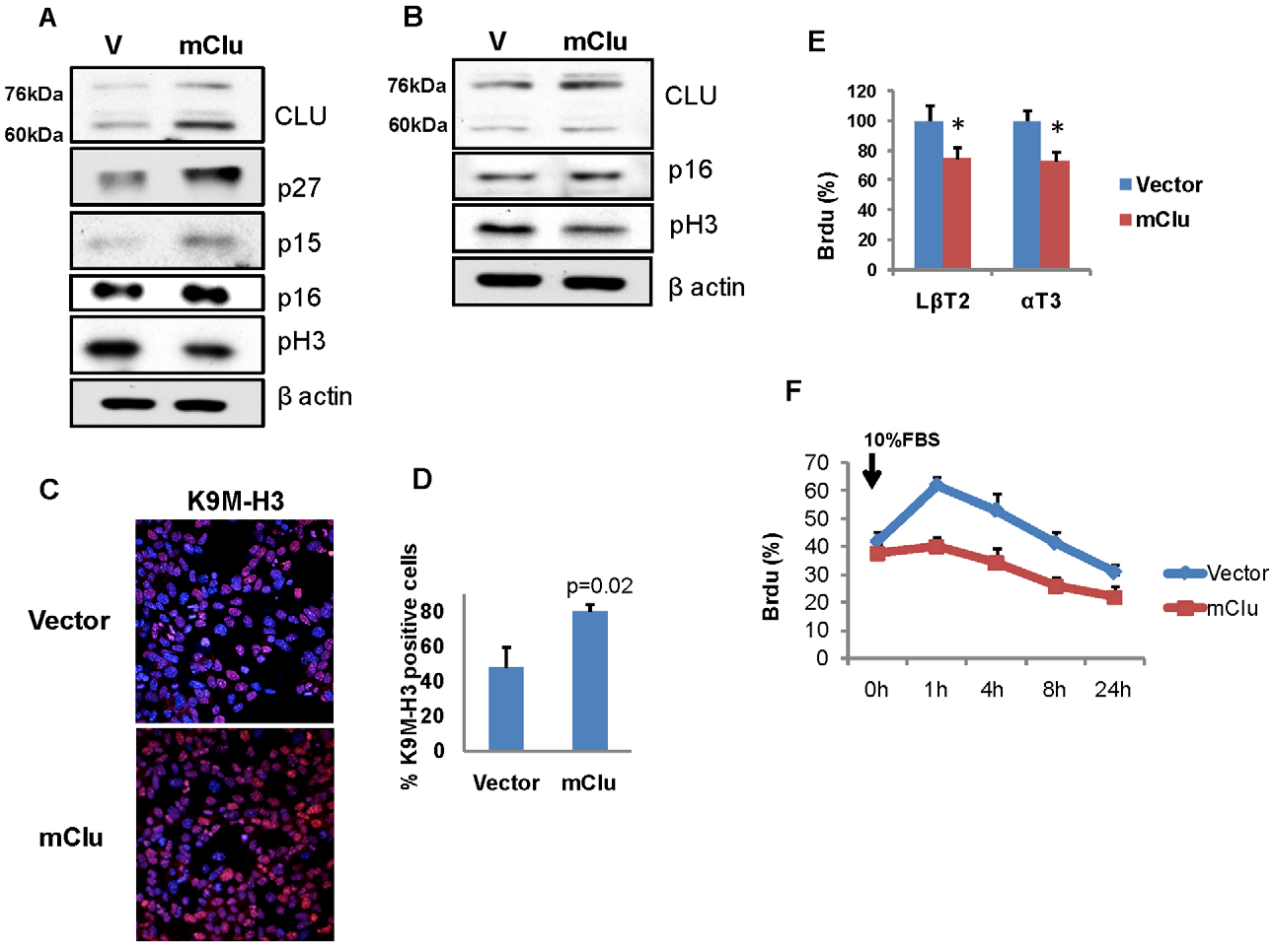
Clusterin restrains pituitary cell proliferation by inducing Cdk inhibitors. Western blot analysis of Cdk inhibitors and proliferation markers **A**) in LβT2 cells, **B**) in αT3 cells 48 h after transfection with m*Clu*; **C**) Confocal images of immunofluoprescence of histone H3 methylation on lysine 9 (H3-K9M) (red) in vector and *Clu*-expressing αT3 cells 48 hours after transfection; **D**) Quantification of positive H3-K9M foci. Cells were fixed, stained with H3-K9M antibody, and one thousand cells/field counted in three randomly chosen visual fields; **E**) Percentage of BrdU positive cells 48 h after transfection with m*Clu*. Triplicate samples were pulsed with BrdU for 30 min and analyzed by flow cytometry, *, p<0.05; **F**) αT3 cells stably overexpressing *mClu* or vector were synchronized in 0.1% fetal bovine serum for 18 hours, and then cultured in 10% fetal bovine serum. At the indicated times, duplicate samples were pulsed with BrdU for 30 min, analyzed by flow cytometry, and cells in S-phase identified by staining with BrdU antibodies.

As high clusterin expression was observed in benign gonadotroph adenomas and in small slow-growing αGSU.*PTTG* pituitary tumors (Figures 3 and 4E), and also in LβT2 cells overexpressing m*Pttg* (Figure 5A,B), we analyzed the effects of altering intracellular clusterin levels. Transient transfection with m*Clu*-pIRES2-ZsGreen1 resulted in increased p15, p16, and p27 expression in LβT2, and p16 in the αT3 cell line. In contrast, levels of phosphorylated histone H3 (pH 3), a specific marker for S and M phases were attenuated in both cell types after clusterin transfection (Figure 7A,B).

Senescence-associated heterochromatic foci (SAHF) mark concentrated spots of transcriptionally silenced DNA [44]. This localization is accompanied by focal accumulation of specific heterochromatin-associated modified histone. Methylation of lysine 9 of histone H3 (K9M-H3) [8], [44] is associated with restraint of cellular proliferation [44]. Using a specific antibody in LβT2 cells transiently transfected with m*Clu*, we observed 30% increase in H3-K9M-positive cells (Figure 7 C,D), concordant with decreased murine gonadotroph cell proliferation observed when clusterin was overexpressed (Figure 7E).

After synchronization of αT3 cells stably overexpressing m*Clu*, and adding 10% FBS, cells were pulsed with BrdU, and flow cytometry demonstrated that BrdU incorporation was decreased, reflecting attenuated DNA synthesis (Figure 7F).

**Figure 8:**
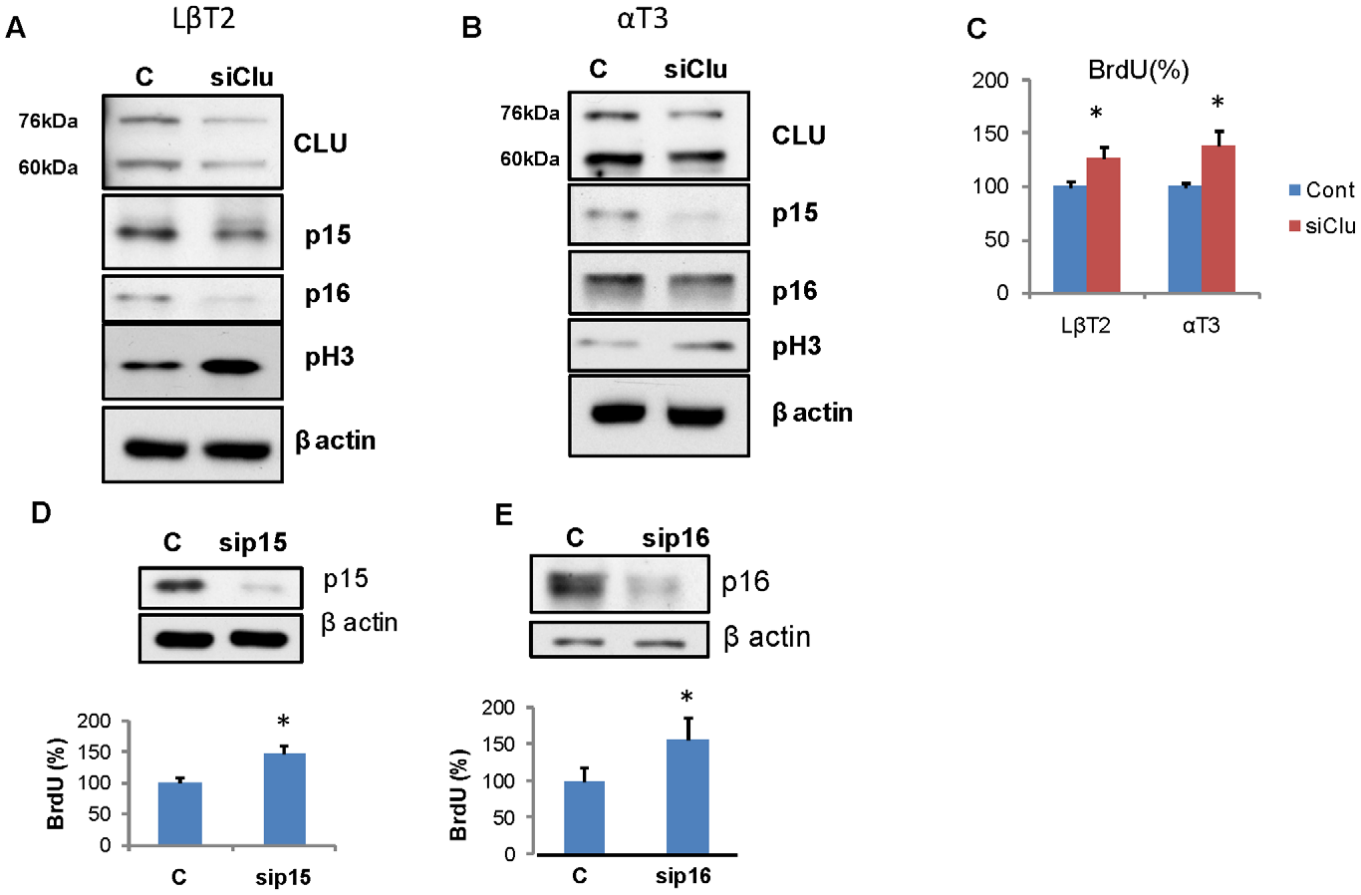
Clusterin attenuation promotes proliferation. Western blot analysis of Cdk inhibitors and proliferation markers **A**) in LβT2 cells, **B**) in αT3 cells; **C**) Percentage of BrdU positive cells 48 h after transfection with si*Clu*. **D**) Upper panel, Western blot confirms p15 down-regulation, Lower panel, Percentage of BrdU positive LβT2 cells 48 h after transfection with sip15. **E**) Upper panel, Western blot confirms p16 down-regulation, Lower panel, Percentage of BrdU positive LβT2 cells 48 h after transfection with sip16. For BrdU detection, cells were fixed, stained with BrdU antibody and one thousand cells/field in three randomly chosen fields counted. *, p<0.05.

Next we suppressed clusterin expression in LβT2 m*Pttg* stable transfectants, and also tested αT3 cells, where endogenous clusterin levels were relatively high. Both cell lines were transfected with 6 nM of two different siRNAs directed against clusterin, and the pRS sh*Clu*-GFP RNA expressing plasmid. Treatments with siClu#2 and transfection with sh*Clu*-GFP both depleted clusterin mRNA by 80% as measured by real time PCR (data not shown). Figure 8 (A,B) shows Western blots of representative experiments using siClu#2 where both clusterin protein forms were down-regulated, p15 and p16 levels were low, and pH 3 protein levels induced in both cell lines. In LβT2m*Pttg* cells and in αT3 cells, clusterin suppression led to increased numbers of cells incorporating BrdU respectively (p<0.05) (Figure 8C). Depletion of clusterin by sh*Clu* RNA resulted in 30 and 26% increase in BrdU-positive LβT2m*Pttg* cells and αT3 cells respectively (data not shown).

The results show that in pituitary gonadotroph cells, induced clusterin restrains proliferation associated with up-regulated p15 and p16, while clusterin depletion led to decreased p15 and p16, accompanied by increased cell proliferation. Indeed, suppression of p15 and p16 transcription by respective siRNAs markedly increased the number of BrdU-incorporated LβT2m*Pttg* cells 48 hours after transfection, reflective of increased cell proliferation (Figure 8 D,E).

### FOXL2 activates clusterin promoter in gonadotroph pituitary cells

**Figure 9:**
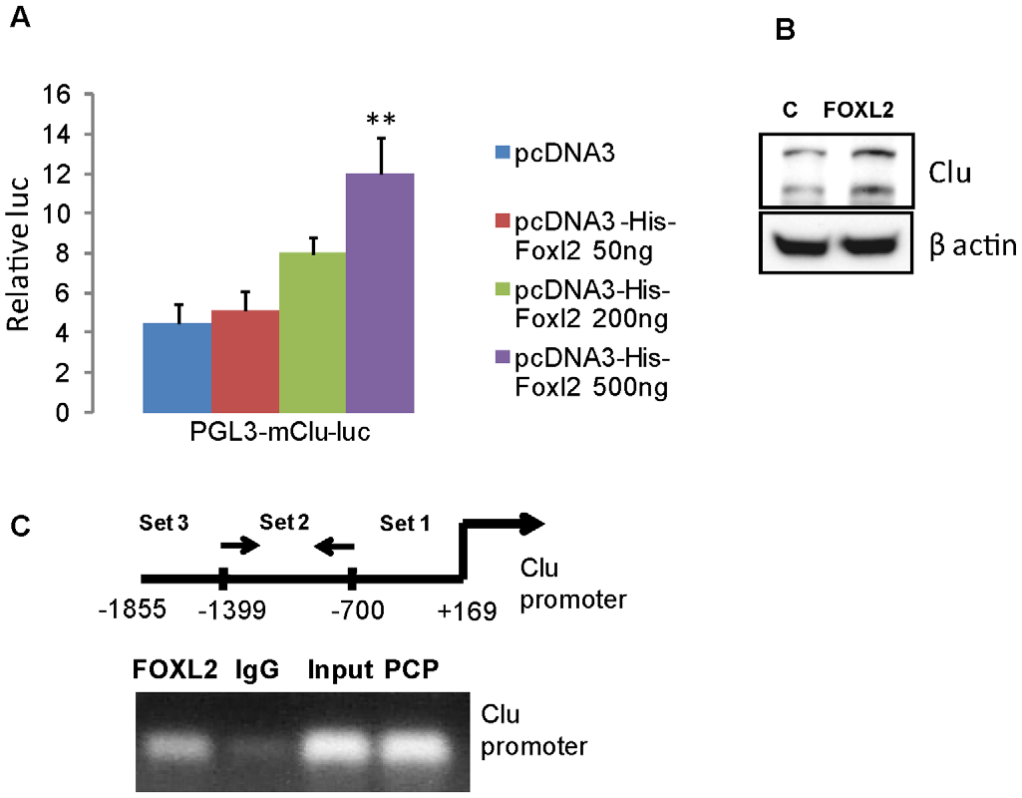
FOXL2 stimulates the clusterin promoter. **A**) Effects of FOXL2 on the clusterin promoter in αT3 cells 24 h after transfection. Cells were co-transfected with 200 ng murine pGL3-luc-m*Clu* reporter plasmid and indicated amounts of pcDNA3-His-mFoxl2. The ratio of luciferase to co-trasfected β-galactosidase control reporter vector was normalized to pCDNA3-null expression vector. SEM was calculated from triplicate assays, and experiments repeated three times with similar results. Results of a representative experiment are shown; **,p<0.01; **B**) Western blot analysis of clusterin expression in αT3 cells 24 hours after transfection with pcDNA3-His-mFoxl2; **C**) ChiP assay was performed in nuclear fractions derived from αT3 cell lysates. Top, schematic of the approximate location of primers used in the PCR reactions. Enrichment of specific clusterin promoter sequences was obtained with primer Set 2. FOXL2, specific antibody, IgG, nonspecific antibody, PCP, positive control primers. The experiment was repeated twice, and results of a representative assay shown.

Forkhead box gene transcription factor L2 (FOXL2) is a cell-specific factor for pituitary gonadotroph differentiation and triggers αGSU expression [35]. FOXL2 is abundantly expressed in human pituitary gonadotroph and null cell adenomas [45], and in normal pituitary co-localizes with LH, FSH and αGSU [35]. As distribution of FOXL2 appeared to mirror clusterin expression in pituitary adenomas, we tested whether FOXL2 stimulates clusterin in gonadotroph cells. pGL3-m*Clu*-luc reporter plasmid was co-transfected with the murine FOXL2 (pcDNA3-His-*Foxl2*) in αT3 cells (Figure 9A), and luciferase activity was induced∼3 fold (p<0.01) indicating the stimulatory effect of FOXL2 on the clusterin promoter. Clusterin protein levels were also enhanced in cells transfected with pcDNA3-His-*Foxl2* (Figure 9B). In contrast, clusterin was not induced in ACTH-secreting AtT20 murine corticotroph pituitary cells transfected with pcDNA3-His-*Foxl2* (data not shown), indicating cell specificity of FOXL2 action on the clusterin promoter.

We then tested whether FOXL2 is recruited to the endogenous clusterin promoter. Lysates derived from αT3 pituitary gonadotrophs were isolated and chromatin immunoprecipitation (ChiP) assays performed with a polyclonal FOXL2 antibody. FOXL2 was shown to bind the clusterin promoter, spanning −700–1339 nucleotides upstream from the transcription start site (Figure 9C), but did not bind the −1468–1865 or +1–700 promoter regions (not shown). Enrichment of specific −700–1339 clusterin promoter sequences in the precipitate indicated FOXL2 association with the clusterin promoter in vivo.

## Discussion

Pituitary lineage specificity determines highly distinct peripheral pituitary trophic hormone actions as exemplified by differentiated GH, PRL, ACTH, TSH or gonadotroph hormone functions. Cell-specific pituitary hormone synthesis is regulated by specific hypothalamic, intrapituitary and peripheral hormone signals [46], [47], [48], [49], [50]. Pituitary cells are also sensitive to aneuploidy, DNA damage or oncogene overexpression. In response to these insults, we show here that cell-type specific trophic pathways are activated, with the common end-point of pituitary cell proliferation arrest.

GH secreting tumors exhibit high PTTG, features of aneuplody, chromosomal instability, activation of DNA damage responses, p21-dependent cell proliferation restraint and senescence [34]. Although high PTTG levels are also observed in gonadotroph pituitary adenomas [26], unlike GH-cell adenomas, we now show that gonadotroph adenomas do not express p21, but abundantly express clusterin in a cell specific manner. In contrast, only modest clusterin expression was observed in non-tumorous pituitary glands and in GH/PRL- secreting tumors, while clusterin was undetectable in 76% of the very rarely encountered pituitary carcinomas.

The results in human tumors were validated using *in vivo* and *in vitro* models of gonadotroph cell adenomas with high PTTG expression. Reflecting pro-proliferative properties of excess PTTG, αGSU.*PTTG* pituitary glands exhibit microadenoma formation [38], also evidenced by increased *in vivo* pituitary BrdU incorporation, and up-regulated proliferation markers including PCNA and E2F1. Similar to human pituitary adenomas, in the αGSU.*PTTG* pituitary gland, high PTTG levels result in aneuplody and chromosomal instability, also evidenced by DNA damage, pATM induction and activation of p53/p27 pathways known to arrest cell proliferation in the course of continuing DNA damage [39]. Concordantly, both the pre-tumorous αGSU.*PTTG* pituitary gland and pituitary tumors express high levels of clusterin, p15 and p16.

The results suggest a biphasic response to transforming effects of excess PTTG *in vivo*: Abundant PTTG is apparently sufficient to trigger an initial proliferative burst leading to hyperplasia and tumor initiation, however, inability of these pituitary tumors to undergo persistent further growth, is likely due to proliferation-restraining pathways activated by PTTG overexpression. These finding underscore the observed SA-β-galactosidase activation in pre-tumorous αGSU.*PTTG* glands and in LβT2 cells constitutively expressing *Pttg*.

High clusterin levels were observed in the αGSU.*PTTG* pituitary gland and in LβT2 cells stably and transiently transfected with m*Pttg*, suggesting that in human gonadotroph adenomas clusterin might be also induced by high PTTG levels. In gonadotroph cells, PTTG-overexpression is accompanied by C/EBP induction. C/EBP proteins uniquely regulate cell-type specific growth and differentiation [41], [42]. C/EBPβ is associated with oncogene-induced senescence [51], while C/EBPδ triggers growth arrest and cell differentiation [43], [52], [53]. Both C/EBPβ and C/EBPδ are shown here to activate the clusterin promoter, and induced clusterin protein expression was evident in gonadotroph cells and pituitary tissue overexpressing PTTG. Furthermore, a specific gonadotroph cell lineage transcription factor FOXL2, independently activates the clusterin promoter in these cells.

We show that forced clusterin expression in LβT2 and αT3 pituitary gonadotroph cells triggers a linage-specific cytostatic response, inducing p15, p16, or p27; decreased cell proliferation was also evidenced by lower expression of pH 3, similar to observations in prostate cancer cells [54]. Accordingly, when either p15 or p16 gene expression were suppressed, pituitary cell proliferation was enhanced. These results are in accordance with those showing that TGFβ-induced p15 decreases proliferation and induces cell cycle arrest in rat GH_3_ pituitary cells [55]. Thus, clusterin-triggered p15 and p16 likely restrain pituitary cell proliferation in αGSU.*PTTG* pituitary tumors and in LβT2 gonadotroph-derived cells. Induced p15 in human gonadotroph adenomas might therefore limit growth of these tumors.

Custerin function in tumorigenesis is unclear. Clusterin expression is enhanced in human prostate cancer, and antisense oligonucleotides targeting clusterin inhibit prostate tumorigenesis [56]. Clusterin also induces breast cancer cell growth and metastatic progression [57] and is associated with human lung adenocarcinoma cell growth [58]. The nuclear anti-apoptotic form of clusterin is induced in late stage cancers following chemotherapy, hormonal ablation or radiotherapy, thus protecting tumor cells undergoing damaging stress [59]. As pituitary carcinomas are rarely treated with radiation or chemotherapy before surgery, we did not observe clusterin expression in human carcinoma specimens, as expected. Several lines of evidence also point to the role of clusterin as a tumor suppressor protein. Thus, clusterin was down-regulated, and its expression inversely proportional to tumor grade/or metastatic stage [16], [18], [60]. Patients with clusterin-positive lung cancer have enhanced disease-free survival [61]. Moreover, *Clu^−/−^* mice are more prone to oncogene-induced tumorigenesis [17], [21]. Although clusterin restrains proliferation of untransformed epithelial cells [21] and acts primarily as a tumor-suppressor during early stages of carcinogenesis [16], [21], when re-expressed in advanced cancers, clusterin might promote tumor growth.

Based on the results presented here, we propose that in benign pituitary tumor cells of gonadotroph origin, the role of clusterin is to restrain proliferation. Similar affects were demonstrated for TGFβ1, which functions as a tumor suppressor in normal epithelial cells and during early stages of tumor development [62]. In late-stage tumors TGFβ1 exhibits features of a tumor promoter, modulating vascular and immune compartments of the tumor stroma [63]. Similarly, in normal fibroblasts [32] and in benign pituitary tumor cells (here and [34]), high PTTG restrains the cell cycle and leads to senescence. In transformed tumor cell lines and in malignant tumors, overexpressed PTTG triggers production of FGF-2 and VEGF-A, cell cycle progression and angiogenesis, and in malignant tumors, high PTTG levels correlate with tumor invasiveness and serve as a marker of poor prognosis (reviewed in [26]). Thus, the cellular environment appears to determine end-point effects of these proteins.

**Figure 10:**
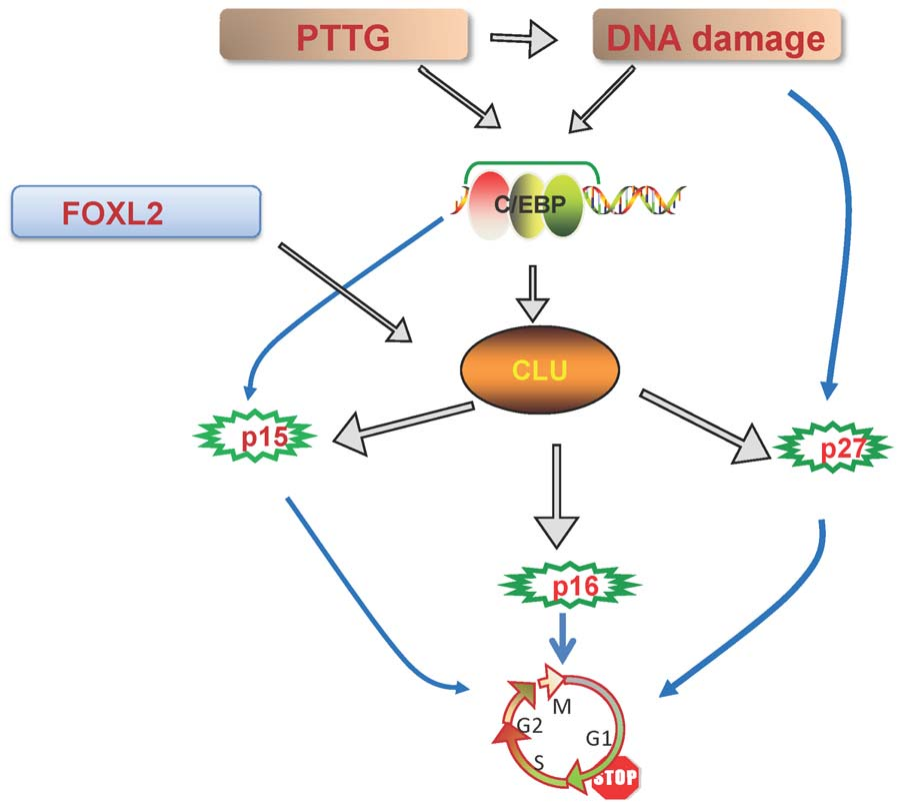
Proliferation restricting pathways in the pituitary gonadotroph cell lineage. FOXL2 directly activates the clusterin promoter, while *Pttg* overexpression results in proliferation, DNA damage and stimulation of C/EBPβ and δ; C/EBPs activate the clusterin promoter. High levels of secretory clusterin trigger expression of Cdk inhibitors p15, p16 and p27, and C/EBPβ also cooperates to induce p15. Up-regulated tumor suppressor proteins likely underlie proliferation restraint preventing uncontrolled growth of benign pituitary adenomas of gonadotroph cell origin.

Our hypothesis is outlined in Figure 10. Human gonadotroph tumors express both FOXL2 and PTTG. The results shown here indicate that FOXL2 directly activates the clusterin promoter, while PTTG triggers clusterin via C/EBPs. Both C/EBPs and clusterin are also induced by DNA damage (data not shown and [59]). High clusterin, in turn, provokes p15, p16 and p27 expression *in vivo* and cell-specifically *in vitro* thus restraining gonadotroph cell proliferation. These observations point to the existence of intrinsic lineage-specific pathways restricting pituitary cell cycle progression. Activation of these pathways should be considered as a contributing factor underlying the overwhelmingly benign nature of pituitary adenomas, enabling maintenance of vital pituitary homeostatic and metabolic functions, while protecting the hormone-secreting gland from destruction by malignancy.

## Methods

### Human tissue samples

Pituitary tumors were freshly collected at transsphenoidal surgery according to an approved Cedars Sinai and Mayo Clinic Institutional Review Board protocols. Written informed consent were obtained from all participants. Samples were formalin-fixed and paraffin-embedded for immunohistochemistry. Diagnosis of individual tumors was established on the basis of clinical features, histology, and pituitary hormone immunohistochemistry. Non-functioning tumors exhibiting gonadotroph-cell markers, including alpha-glycoprotein subunit (αGSU), LH or FSH were selected for study. Normal anterior pituitary tissue controls were freshly obtained at surgery.

### Animals

Experiments were approved by the Cedars Sinai Institutional Animal Care and Use Committee (protocol # 2683). Mice in a B6C3 genetic background harboring the αGSU-*PTTG1*-IRES-eGFP (αGSU.*PTTG*) transgene were previously described [28]. To obtain WT and αGSU.*PTTG* mice from the same breeding, we crossbred αGSU.*PTTG^+/−^* males and females, and genotyped by PCR.

### BrdU incorporation in vivo

Mice were injected i.p. with BrdU (50 µg/g BW, Sigma-Aldrich, St Louis, MO) three times at 3 hour intervals, sacrificed 24 h after the first injection and pituitary sections stained (5-BrdU Labeling and Detection Kit, Roche, Palo Alto, CA). Three randomly chosen visual fields (1000 cells/per field) were counted, and three sections/per animal were derived from three of each genotype analyzed.

### SA-β-galactosidase activity

Senescence-associated (SA)-β-galactosidase enzymatic activity was detected in pituitary cryosections (10 µm) using a β-galactosidase staining kit (Senescence Cell Staining Kit, Sigma-Aldrich). Only senescent cells stain at pH 6.0. SA-β-galactosidase activity *in vitro* was assayed in 6-well plates in triplicate [29]. Three randomly chosen visual fields/per well were identified, and 1000 cells/per field counted.

### Protein analysis

Pituitary tissues or cells were processed (Immunoprecipitation Kit, Roche Diagnostics, Indianapolis, IN) for Western blot analysis, proteins separated by SDS-PAGE, electroblotted onto Millipore membranes (Millipore, Temecula, CA), and incubated overnight with antibodies, followed by corresponding secondary antibodies (Sigma-Aldrich, St. Louis, MO). Antibodies purchased from Santa Cruz (CA): PCNA, E2F1; and MSH2, MLH1, Rad51, p27, p16, Clu. PTTG, Ki67, p53, C/EBPβ, C/EBPδ were obtained from Abcam (Cambridge, MA). We also used antibodies to p15 (Biosource); phosphoHistone 3 (ser10) (Cell Signaling Technology, Danvers, MA), and β actin (Sigma-Aldrich, St. Louis, MO).

For immunofluorescence analysis of human tissue we used antibodies to γH2A.X (ser139), CLU and H3-K9M antibody, all from Millipore (Billerica, MA); phosphoATM (ser1981, Upstate Biotech (Millipore); p15, p16, all from Abcam; and p21 (Cell Signaling) antibodies followed by corresponding secondary antibodies conjugated with Alexa 488 or with Alexa 568 fluorescent dye (both Molecular Probe, Carlsbad, California). Antigen retrieval was performed in 10 mM sodium citrate, and control reactions were devoid of primary antibodies or stained with blocking antibodies. Samples were imaged with a Leica TCS/SP spectral confocal scanner (Leica Microsystems, Mannheim, Germany) in dual emission mode to distinguish autofluorescence from specific staining.

Human pituitary adenoma PTTG was detected by immunohistochemistry with the same antibodies as for Western blot, and using an avidin-biotin-peroxidase kit (Vector Laboratories, Burlingame, CA).

### Cells, constructs, plasmids and transfections

Mouse pituitary gonadotroph LβT2 and αT3 cell lines were generously provided by Dr. Pamela Mellon (UC San Diego). These cells, immortalized with SV40 T-antigen [64], are the only functional gonadotroph cell lines available.

Murine testis mRNA was used as a source for *Pttg1*. Primers were designed as follows: forward, GGAATTCCATGGCTACTCTTATCTT, reverse CGGGATCCCCGAATATCTGCATCGT. The Expand High Fidelity PCR system (Roche Diagnostics) was used for amplification reactions. PCR products were double digested (EcoR1/BamH1), purified and ligated (DNA ligation Kit, Takara Bio, Japan) into pIRES2-ZsGreen1 vector (Clontech, Mountain View, CA) to generate cells that co-express *Pttg* and a ZsGreen tag.

Mouse clusterin expressing plasmids were amplified from pCMv6-m*Clu* (Origene, Rockville, MD) by using TaKaRa LA Taq (Takara), and cloned into the pIRES2-ZsGreen1 vector (Clontech). The following primers were used for PCR: clusterin forward: 5′ CGGAATTCATGAAGATTCTCCTGCTGTGCG T 3′; reverse: 5′ CGG GAT CCTCATTCCGCACGGCTTTTCCT 3′.

Mouse clusterin promoter fragment (−1855 to +169) was amplified from mouse genomic DNA using TaKaRa LA Taq, inserted into the pGL3-Basic luciferase reporter vector (Promega, San Luis Obispo, CA) and the following primers used for PCR: forward 5′ GGG GTA CCA CAT TCC TCC AAG TTT CTG 3′, and reverse 5′ CGG GAT CCA TGG GCT CTA GTC ACC TC 3′.

LβT2 and αT3 cells were stably transfected with m*Pttg*-pIRES2-ZsGreen1 or with *mClu-pIRES2-ZsGreen1* to create LβT2m*Pttg* cells or αT3m*Clu* cells respectively, or with pZsGreen1-N1 alone (vector). Cells were grown in the presence of 400 µg/ml geneticin (Invitrogen, Carlsbad, CA). Fourth- and fifth-generation enriched stable LβT2m*Pttg* or αT3m*Clu* or vector cells were used for experiments.

Short hairpin RNA expressing vector targeting murine clusterin shClu-pGFP-V-RS (sh*Clu*) were purchased from OriGene( Rockvillle, MD). Small interfering RNAs targeting murine clusterin, p15 and p16 (si*Clu*, 1 and 2; sip15, 1 and 2; sip16, 1 and 2) and scrambled siRNAs as negative controls are from Ambion (Foster City, CA). Small interfering RNAs targeting murine C/EBPβ, C/EBPδ and scrambled siRNA are from Qiagen (Valencia, CA). Murine pcDNA3.1(−)C/EBPβ was from Addgene (plasmid12557), and murine pcDNA3 C/EBPδ was a generous gift from Dr. Koeffler (Cedars Sinai Medical Center). As fetal bovine serum contains clusterin, cell transfections with m*Clu*, sh*Clu* and si*Clu* were conducted in 0.5% fetal bovine serum (FBS). Transfections were performed using Lipofectamine 2000 (Invitrogen,Carlsbad, CA).

pcDNA3-His-mFoxl2 overexpressing plasmid was a generous gift from Dr. Wei-Hsiung Yang (Mercer University School of Medicine, Savannah, GA) with the permission of Dr. Buffy S. Ellsworth (Southern Illinois University School of Medicine, Carbondale,IL).

### Luciferase Assays

LβT2 and αT3 cells were transfected with 800 ng pGL3-luc basic vector or pGL3-luc-m*Clu* reporter plasmid and co-transfected with pcDNA3 or pcDNA3 encoding murine C/EBPβ or δ in 12-well plates. Twenty four later cells were harvested and monitored for luciferase activity (Promega, Madison, WI). Light emission was evaluated by luminometer, and normalized to a β-galactosidase luciferase reference plasmid.

### Chromatin Immunoprecipitation Assay *(ChiP)*

ChiP was performed (Chip-IT Express kit, Active Motif, Carlsbad, CA) using αT3 pituitary gonadotroph cells which exhibit abundant clusterin expression. Cells were cross-linked with formaldehyde, harvested, sonicated, nuclear fraction isolated, and chromatin immunoprecipitation performed with polyclonal FOXL2 antibody (Abcam) as well as non-specific mouse IgG. DNA released from precipitated complexes was amplified by PCR with 3 pairs of specific primers spanning 1855 nucleotides upstream from the murine clusterin transcription start site. Primer set 1 (+169–700): F 5′CTTTCCTACCCCAGCGCCGC-3′,R 5′-ACCCTGCGCAGCTTTCCACC-3′; Primer set 2 (−700–1399): F 5′-GGACAGCAGAGGCCTTCGGGA-3′,R 5′-GGGCTGCTTGCTGGTCCCTTG-3′; Primer set 3 (−1400–1855): F 5′-AGGCCCAAGGGCAGAGTGGT-3′, R 5′-ACACAGTTTGGGTGGCAGGCC-3′. PCR products were resolved in 1% agarose gel and visualized by ethidium bromide.

### Apoptosis

The rate of apoptosis was assessed *in vitro* using the *In Situ* Cell Death Detection Kit, AP (TUNEL assay, Roche Diagnostics, Indianapolis, IN). Three randomly chosen visual fields, each containing 1000 cells were counted.

### Cell proliferation assay

Asynchronized cells were pulsed with 10 µM BrdU (Sigma, St Louis, Missouri) in phosphate buffered saline for 30 min at 37°C. Cells were washed, harvested, fixed in 75% ethanol/50 mM glycin at pH 2.0 and analyzed by FACScan (Becton Dickinson, Mountain View, California). Experiments were performed in triplicate. In separate experiments αT3*mClu* or αT3vector cells were synchronized in 0.1% FBS for 18 hours, and then stimulated by addition of 10% FBS. At the indicated times, duplicate samples were pulsed with BrdU for 30 min, analyzed by flow cytometry, and S-phase cells identified by staining with BrdU antibodies.

### Statistics

Clusterin and p15 protein expression were compared between human GH/PRL- and gonadotroph cell pituitary tumors using Wilcoxon Rank Sum Test. Cellular labeling indices were analyzed using ANOVA followed by non-parametric t-test (Mann-Whitney) or Student t-test. Probability of *p*<0.05 was considered significant.
